# Supplementary material for: Gravity compensation for leachate grid cleaning robots in waste-to-energy plants: A modeling and simulation study
Source: PLoS One. 2026 May 19;21(5):e0349592. doi: 10.1371/journal.pone.0349592 (PMC13186384; doi:10.1371/journal.pone.0349592)
Supplement: Attachment — Submitted filename: Response to Reviewers.docx [file pone.0349592.s002.docx]

Response to reviewers

Dear Academic Editor: Shamshad Alam, PhD, Dear reviewers

We gratefully thank the Editor and all Reviewers for their time spent making constructive remarks and useful suggestions, which have significantly improved the quality of the manuscript. Each suggested revision and comment brought forward by the reviewers was accurately incorporated and considered. The Reviewers' comments are addressed below point by point with corresponding revisions.

Academic Editor

**1.Comment:**Please ensure that you refer to Table 8 in your text as, if accepted, production will need this reference to link the reader to the Table.

**1. Reply:** Thank you for the reminder. We have added an explicit reference to Table 8 in Section 4.3. The modification is as follows:

Section 4.3 – Added reference to Table 8

Original text:

The resulting positioning errors were recorded and compared to the no-contact case.

Revised text:

The resulting positioning errors were recorded and compared to the no-contact case. These results are summarized in Table 8.

This change ensures that Table 8 is properly cited in the text, linking readers to the data. The modification is highlighted in the revised manuscript.

**2.Comment:**In the online submission form, you indicated that The data supporting this study's findings are available from the corresponding author upon reasonable request..

All PLOS journals now require all data underlying the findings described in their manuscript to be freely available to other researchers, either

1. In a public repository,

2. Within the manuscript itself, or

3. Uploaded as supplementary information.

This policy applies to all data except where public deposition would breach compliance with the protocol approved by your research ethics board. If your data cannot be made publicly available for ethical or legal reasons (e.g., public availability would compromise patient privacy), please explain your reasons on resubmission and your exemption request will be escalated for approval.

**2. Reply:** Thank you for the reminder regarding PLOS data availability policy. We confirm that all data underlying the findings in our manuscript are included within the paper itself. Therefore, we have revised the Data Availability Statement accordingly in Cover letter.

Original:

The data supporting this study's findings are available from the corresponding author upon reasonable request.

Revised:

We confirm that our submission contains all raw data required to replicate the results of our study.

**3.Comment:**Thank you for stating in your Funding Statement:

“This work was supported by the Henan Province Science and Technology Research Project(No.252102220033), Natural Science Foundation of Henan（No.252300420072） and the education department of Henan Province (No. 24A460023 and No. 26A460029).”

Please provide an amended statement that declares *all* the funding or sources of support (whether external or internal to your organization) received during this study, as detailed online in our guide for authors at http://journals.plos.org/plosone/s/submit-now. Please also include the statement “There was no additional external funding received for this study.” in your updated Funding Statement.

Please include your amended Funding Statement within your cover letter. We will change the online submission form on your behalf.

**3. Reply:** Thank you for the instruction. We have revised the Funding Statement as requested. The amended statement will be included in the cover letter as follows:

Cover Letter – Revised Funding Statement

This work was supported by the Henan Province Science and Technology Research Project (No.252102220033), Natural Science Foundation of Henan (No.252300420072), and the Education Department of Henan Province (No. 24A460023 and No. 26A460029).

There was no additional external funding received for this study.

The funders had no role in study design, data collection and analysis, decision to publish, or preparation of the manuscript.

We understand that the online submission form will be updated accordingly by the editorial office. Thank you for handling this change.

**4.Comment:**Thank you for stating the following financial disclosure:

“This work was supported by the Henan Province Science and Technology Research Project(No.252102220033), Natural Science Foundation of Henan（No.252300420072） and the education department of Henan Province (No. 24A460023 and No. 26A460029).”

Please state what role the funders took in the study. If the funders had no role, please state: "The funders had no role in study design, data collection and analysis, decision to publish, or preparation of the manuscript."

If this statement is not correct you must amend it as needed.

Please include this amended Role of Funder statement in your cover letter; we will change the online submission form on your behalf.

**4. Reply:** Thank you for the instruction. We have revised the Acknowledgments as requested. The amended statement will be included in the cover letter as follows:

Cover Letter – Revised Acknowledgments

This work was supported by the Henan Province Science and Technology Research Project (No.252102220033), Natural Science Foundation of Henan (No.252300420072), and the Education Department of Henan Province (No. 24A460023 and No. 26A460029).

There was no additional external funding received for this study.

The funders had no role in study design, data collection and analysis, decision to publish, or preparation of the manuscript.

We understand that the online submission form will be updated accordingly by the editorial office. Thank you for handling this change.

**5.Comment:**We note that Figures 1, 4, 5 and 8 in your submission contain copyrighted images. All PLOS content is published under the Creative Commons Attribution License (CC BY 4.0), which means that the manuscript, images, and Supporting Information files will be freely available online, and any third party is permitted to access, download, copy, distribute, and use these materials in any way, even commercially, with proper attribution. For more information, see our copyright guidelines: http://journals.plos.org/plosone/s/licenses-and-copyright.

**5. Reply:** Thank you for your query regarding the images in our submission.

We confirm that all figures mentioned are original works created by the authors and do not contain any third-party copyrighted material:

Figure 1 consists of photographs taken by us at a waste-to-energy plant.

Figures 4, 5, and 8 are original diagrams and schematics we created to illustrate our robot design and analysis.

Therefore, no permissions are required for their publication under the CC BY 4.0 license. We trust this meets your requirements.

Reviewer 1

Comments to the Author

This manuscript addresses a relevant engineering problem with a technically sound methodology. While the work is limited to simulation, it provides a solid theoretical foundation for future physical implementation. The writing is clear and the results are promising. I recommend minor revision to address the concerns outlined in the following, particularly regarding validation limitations and parameter documentation:

**1.Comment:**Expand validation discussion:Clarify that results are simulation-only and discuss implications for real-world implementation.

**1. Reply:** Thank you for this valuable suggestion. We have revised the manuscript to explicitly state that the validation is simulation-based and to provide a thorough discussion of the challenges and necessary steps for real-world implementation. The specific modifications are as follows:

(1). Abstract (last two sentences)

Original:

This work provides a validated solution for high-precision robotics in hazardous environments.This model-based compensation strategy provides a generalizable solution for precision control of long-reach hydraulic manipulators operating under heavy gravitational loads.

Revised:

This model-based compensation strategy provides a generalizable theoretical framework for precision control of long-reach hydraulic manipulators. The current study is validated through high-fidelity Adams simulations, achieving a simulated repeatable positioning accuracy of ±0.1 mm. However, it is essential to emphasize that these results represent an idealized upper bound. Real-world hydraulic systems introduce complexities not captured in simulation—including backlash, nonlinear friction, valve dynamics, hysteresis, leakage, and sensor quantization—all of which will degrade practical accuracy. Therefore, this work establishes a theoretical foundation requiring experimental validation on physical hardware. Future work will focus on physical prototyping and on-site testing to address real-world challenges.

(2). Introduction (end of Section 1)

Original (third contribution):

3) Validated the strategy via Adams simulations, achieving ±0.1 mm repeatable accuracy (exceeding the 0.5 mm engineering requirement).

Revised:

3) Theoretically validated the strategy via Adams simulations, achieving ±0.1 mm repeatable accuracy (exceeding the 0.5 mm engineering requirement) and providing a robust foundation for real-world deployment.

(3). Section 4 (Gravity Error Simulation Experiment) – first paragraph

Original:

The robot's mechanical model was imported into Adams software. Gravity was applied. ...

Revised (addition at the beginning):

To rigorously evaluate the proposed feedforward compensation strategy prior to physical prototyping, a high-fidelity virtual prototype was developed using Adams multibody dynamics software. The robot's mechanical model was imported into Adams software. Gravity was applied. ... This simulation-based validation allows for an idealised assessment of the compensation model's theoretical efficacy by isolating gravitational effects from other real-world disturbances.

(4). Section 5 (Conclusion) – completely restructured into two subsections

Original section (single block of text) has been replaced with:

5.1 Conclusion

This study addressed the critical challenge of gravity-induced positioning errors in leachate grid cleaning robots via integrated theoretical modeling and Adams multibody dynamics simulations. We proposed a feedforward static compensation strategy centered on an analytical joint stiffness-gravity deformation model, tailored for hydraulically driven weak-rigid manipulators in hazardous leachate corridors. The simulation results demonstrate that the proposed strategy effectively suppresses end-effector pose drift, reducing positioning errors by over 92% in all directions and achieving a repeatable positioning accuracy of ±0.1 mm in a simulated environment. This performance far surpasses the 0.5 mm precision requirement for automated leachate grid cleaning, providing strong theoretical evidence for the method's potential applicability.

5.2 Implications for Real-World Implementation and Future Work

While the simulation results are highly promising, this study is not without limitations. Translating these theoretical findings to a physical robot operating in the harsh environment of a leachate corridor presents several challenges that must be addressed in future work.

**Environmental Robustness:** The current simulation assumes ideal conditions. In reality, temperature fluctuations (0–60°C) in the corridor can alter reducer stiffness (by an estimated ±5% for the JS12/SEA7 units), potentially degrading compensation accuracy over time. Furthermore, the presence of dust, humidity, and corrosive gases may affect joint friction and long-term component reliability.

**Model Fidelity and Disturbances:** The static model does not account for dynamic reaction forces encountered during the actual unclogging process, such as those from compacted debris or viscous leachate adhesion. These forces could introduce additional unmodeled deflections.

**Control System Integration:** Implementing the feedforward model in a real-time controller requires precise measurement of joint angles and computationally efficient execution of the torque and deformation calculations. The potential need for online calibration routines to maintain accuracy over the robot's lifetime must also be investigated.

To bridge the gap between simulation and reality, our future research will focus on three key areas:

1) Physical Prototyping and On-Site Validation: We will fabricate a physical prototype integrating explosion-proof hydraulic components compliant with relevant standards (e.g., GB3836). This prototype will be deployed in a waste-to-energy plant for on-site validation, allowing us to quantify the compensation strategy's true efficacy under real operational conditions and verify its long-term stability.

2)Algorithm Enhancement with Sensor Feedback: To counteract environmental influences, we plan to enhance the compensation algorithm by incorporating real-time temperature sensors for dynamic correction of reducer stiffness variations. Future iterations may also explore hybrid approaches that use the feedforward model for bulk compensation and selective feedback from rare, safe calibration routines to correct for residual errors.

3)Generalization to Other Applications: We will explore the extension of this feedforward strategy to other hydraulically driven manipulators (e.g., 6-DOF arms) for diverse hazardous tasks, such as industrial pipeline unclogging or nuclear facility maintenance, further validating its generalizability.

We believe these revisions directly address your comment by transparently acknowledging the simulation-based nature of the current work and providing a realistic discussion of the path toward practical deployment. All changes are highlighted in the revised manuscript.

**2. Comment:** Strengthen limitations section:Include discussion of factors that could affect real-world performance.

**2. Reply:** Thank you for this important suggestion. We have significantly expanded the limitations discussion in Section 5.2 to provide a more comprehensive analysis of factors that could impact real-world performance. The revised section now systematically addresses environmental, mechanical, and control-related challenges. The specific modifications are as follows:

Section 5.2 (Implications for Real-World Implementation and Future Work) – expanded limitations discussion

Original text (from previous revision):

While the simulation results are highly promising, this study is not without limitations. Translating these theoretical findings to a physical robot operating in the harsh environment of a leachate corridor presents several challenges that must be addressed in future work.

Environmental Robustness: The current simulation assumes ideal conditions. In reality, temperature fluctuations (0–60°C) in the corridor can alter reducer stiffness (by an estimated ±5% for the JS12/SEA7 units), potentially degrading compensation accuracy over time. Furthermore, the presence of dust, humidity, and corrosive gases may affect joint friction and long-term component reliability.

Model Fidelity and Disturbances: The static model does not account for dynamic reaction forces encountered during the actual unclogging process, such as those from compacted debris or viscous leachate adhesion. These forces could introduce additional unmodeled deflections.

Control System Integration: Implementing the feedforward model in a real-time controller requires precise measurement of joint angles and computationally efficient execution of the torque and deformation calculations. The potential need for online calibration routines to maintain accuracy over the robot's lifetime must also be investigated.

Revised and expanded text:

While the simulation results are highly promising, this study has several limitations that must be acknowledged when considering real-world deployment. Translating these theoretical findings to a physical robot operating in the harsh environment of a leachate corridor presents multiple challenges across environmental, mechanical, and control domains.

Environmental Factors:

Temperature Effects: The current simulation assumes constant stiffness at 20°C. In reality, leachate corridors experience temperature fluctuations between 0–60°C due to seasonal variations and exothermic waste decomposition processes. Reducer stiffness (JS12 and SEA7) can vary by an estimated ±5% over this range, as lubricant viscosity changes and housing materials undergo thermal expansion. This stiffness variation would directly affect the accuracy of the deformation model described in Eq. (1), potentially leading to residual positioning errors if not compensated.

Humidity and Corrosive Gases: The corridor atmosphere contains high humidity and corrosive gases (e.g., H₂S, SOₓ, COₓ) resulting from waste fermentation. These conditions may accelerate wear in joint reducers, increase friction, and potentially alter joint stiffness over extended operational periods. The current model does not account for such time-dependent degradation.

Contaminant Accumulation: Dust and leachate residue may accumulate on moving parts, particularly on the rail guidance system and joint seals. This could introduce additional friction torques not present in the simulation, affecting the static equilibrium assumption underlying the torque calculations in Eqs. (3) and (4).

Mechanical and Operational Factors:

Unmodeled Dynamic Loads: The static compensation model assumes quasi-static operation, which is reasonable given the robot's slow cleaning speed (≤10°/s). However, the actual unclogging process involves intermittent contact forces when the drill encounters compacted debris. These impulsive reaction forces, which can reach up to 200 N (the design limit), may cause transient elastic deformations not captured by the static model. Furthermore, the model assumes that joint deformation responds instantaneously to gravitational torque, neglecting any viscoelastic effects in the reducer components.

Link Flexibility: While we justified neglecting link deformation based on the hollow steel structure, under maximum reach configurations (fully extended arm), the combined effect of joint and minor link compliance might become non-negligible. This could introduce second-order positioning errors not accounted for in the current joint-only deformation model.

Grid Deformation Variability: The robot must adapt to deformed grid apertures caused by waste impact. While the single-probe design accommodates this, the compensation model assumes the grid geometry is known. In practice, significant grid deformation may require real-time perception and trajectory adjustment, which the current feedforward approach does not address.

Control and Calibration Challenges:

Initial Calibration Sensitivity: The simulation assumes perfect calibration at the reference pose ([2756, 150, 389] mm). In reality, establishing this calibration requires precise measurement of the end-effector position relative to the grid, which is challenging in the dark, gas-filled corridor environment. Any calibration error would propagate through the compensation model.

Parameter Uncertainty: The joint stiffness values in Table 2 are manufacturer-provided nominal values under ideal conditions. Unit-to-unit manufacturing variations, assembly tolerances, and wear over time introduce uncertainty that could degrade compensation accuracy. The model currently lacks a mechanism to identify or update these parameters online.

Real-Time Implementation Constraints: Implementing the feedforward model requires solving Eqs. (3) and (4) in real-time and adding the calculated error angles to the joint commands. While computationally feasible, this demands precise joint angle feedback and synchronization with the hydraulic servo controllers. Latency in angle measurement or command execution could introduce phase errors between the compensation and actual deformation.

These limitations highlight the gap between idealized simulation and practical deployment. Addressing them requires a systematic approach to physical prototyping and model enhancement, as outlined in the future work roadmap below.

Revised future work roadmap (remainder of Section 5.2):

To bridge the gap between simulation and reality, our future research will focus on three key areas:

(1) Physical Prototyping and On-Site Validation: We will fabricate a physical prototype integrating explosion-proof hydraulic components compliant with relevant standards (e.g., GB3836). This prototype will be deployed in a waste-to-energy plant for on-site validation, allowing us to: (a) quantify the compensation strategy's true efficacy under real operational conditions; (b) measure the actual impact of temperature, humidity, and contaminants on positioning accuracy; and (c) verify long-term stability through extended field trials. The prototype will be instrumented with temperature sensors and joint torque sensors to collect empirical data for model refinement.

(2) Algorithm Enhancement with Sensor Feedback: To counteract environmental influences, we plan to enhance the compensation algorithm in two stages:

Stage 1 – Temperature Compensation: Incorporate real-time temperature sensors at Joints 2 and 3 to dynamically correct reducer stiffness values using empirically derived temperature-stiffness curves, thereby maintaining compensation accuracy across the full 0–60°C range.

Stage 2 – Hybrid Feedforward-Feedback: Explore a hybrid approach where the feedforward model provides bulk compensation, while occasional vision-based measurements of the end-effector relative to grid apertures (during safe, non-cleaning intervals) provide feedback for online calibration of residual errors and model parameter updates.

(3) Generalization and Model Refinement: We will extend the compensation framework to: (a) incorporate dynamic terms for the unclogging contact forces, moving beyond pure static compensation; (b) validate the approach on other hydraulically driven manipulators (e.g., 6-DOF arms) for diverse hazardous tasks such as industrial pipeline unclogging or nuclear facility maintenance; and (c) develop a methodology for in-situ identification of joint stiffness parameters to handle unit-to-unit variations and long-term degradation.

We believe this expanded limitations section provides a more thorough and transparent discussion of the challenges facing real-world implementation, directly addressing your comment. All changes are highlighted in the revised manuscript.

**3. Comment:** Clarify parameter sources: Provide more detail on how stiffness values and other parameters were determined.

**3. Reply:** Thank you for this important observation. We agree that transparency regarding parameter sources is essential for reproducibility and credibility. We have revised the manuscript to explicitly state the origin of all key parameters, including joint stiffness values, geometric dimensions, mass properties, and motion ranges. The specific modifications are as follows:

**Detailed Modifications**

(1) Section 2.2 (Mechanical Design) – end of first paragraph

Original:

Joints 1-4 utilize hydraulic motors coupled with rotary reducers. Joint 5, requiring minimal torque, uses a hydraulic motor for direct drive. The end-effector is driven by a hydraulic motor and RV reducer.

Revised (addition):

Joints 1-4 utilize hydraulic motors coupled with rotary reducers. Joint 5, requiring minimal torque, uses a hydraulic motor for direct drive. The end-effector is driven by a hydraulic motor and RV reducer. All geometric parameters (link lengths, joint offsets, and component dimensions) were obtained directly from the 3D CAD model (SolidWorks 2022) used for the robot design. These values are listed in Table 3 and reflect the actual as-designed configuration.

(2) Section 3.3 (Joint Torsional Deformation Solution) – after Eq. (4)

Original:

Solving joint torsional deformation (Eq. 1, 2) requires joint stiffness ($k$) and joint torque ($\tau$). Joint stiffness is primarily determined by the reducer stiffness for the worm-gear reducers used (motor stiffness contribution is negligible). Reducer stiffness values for Joints 2 and 3 are provided in Table 2.

Revised:

Solving joint torsional deformation (Eq. 1, 2) requires joint stiffness ($k$) and joint torque ($\tau$). Joint stiffness is primarily determined by the reducer stiffness for the worm-gear reducers used (motor stiffness contribution is negligible). The stiffness values for the JS12 (Joint 2) and SEA7 (Joint 3) reducers were obtained from the manufacturer's technical datasheets (Sumitomo Drive Technologies, 2023). These datasheets provide stiffness under rated load conditions at 20°C. It should be noted that these are nominal values; actual stiffness may vary by approximately ±3% due to manufacturing tolerances and assembly conditions. The stiffness values are provided in Table 2.

Additional paragraph added after Table 2:

The geometric parameters (lengths$L_{1}$to$L_{7}$in Eqs. (3) and (4) and Table 3) were directly measured from the 3D CAD model of the robot (SolidWorks 2022). Mass properties ($G_{1}$to$G_{7}$in Table 4) were calculated by the CAD software based on the assigned material densities (structural steel for links, 7,850 kg/m³; hydraulic components, aluminum alloy 6061, 2,700 kg/m³; reducer assemblies, combined density based on manufacturer component masses). The weights in Newtons were obtained by multiplying the calculated masses by gravitational acceleration (9.81 m/s²). Joint motion ranges and maximum speeds (Table 5) were defined based on the operational requirements for grid cleaning and the specifications of the selected hydraulic motors and reducers.

(3) Table 2 – added footnote

Note: Stiffness values are nominal values at 20°C, obtained from manufacturer technical datasheets (Sumitomo Drive Technologies, 2023).

(4) Tables 3 and 4 – added footnotes

Table 3 (Gravity analysis parameters – Lengths, Angles, Torque)

Note: All length parameters (in mm) were measured directly from the 3D CAD model (SolidWorks 2022) of the cleaning robot. $L_{1}$-$L_{3}$ represent link lengths; $L_{4}$-$L_{6}$ represent distances from joint axes to component centers of mass.

Table 4 (Gravity values for components)

Note: Weight values (in N) were calculated from mass properties obtained from the 3D CAD model (SolidWorks 2022). Masses were computed based on assigned material densities: structural steel (7,850 kg/m³) for links, aluminum alloy 6061 (2,700 kg/m³) for hydraulic components, and composite densities for reducer assemblies based on manufacturer-provided component masses. Weights were obtained by multiplying mass by g = 9.81 m/s².

(5) Section 4 (Gravity Error Simulation Experiment) – after trajectory description

Original:

For validating the compensation strategy, a straight-line trajectory is designed to simulate the actual unclogging process of a specific grid aperture (Aperture A at [2756, 150, 389] mm). The trajectory spans from Point B (2656, 150, 389) mm to Point C (2856, 150, 389) mm (Fig. 12)...

Revised (addition):

For validating the compensation strategy, a straight-line trajectory is designed to simulate the actual unclogging process of a specific grid aperture (Aperture A at [2756, 150, 389] mm). The trajectory spans from Point B (2656, 150, 389) mm to Point C (2856, 150, 389) mm (Fig. 12)... This trajectory and the reference aperture position were selected based on the actual grid layout in a typical waste-to-energy plant (refer to Fig. 1) and represent a challenging mid-range reach configuration for the robot. The calibration pose ([2756, 150, 389] mm) was defined as the point where the end-effector is aligned with the target aperture; at this pose, the initial static deformation was zeroed in the uncompensated simulation to replicate the real-world practice of teaching the robot a reference point.

(6) Section 3.3 – additional clarification on stiffness assumptions

New paragraph added before Table 2:

It is important to acknowledge certain assumptions regarding the stiffness parameters. The manufacturer-stated stiffness values represent the torsional stiffness of the reducer output under ideal, purely torsional loading at rated torque. In reality, the presence of combined loads (radial and axial forces) during robot operation may slightly alter the effective stiffness. However, for the long-reach, relatively lightweight manipulator in this study, the gravitational torques dominate and are predominantly torsional about the joint axes; radial and axial loads on the reducers are comparatively small. Therefore, the use of nominal torsional stiffness values is justified for this static compensation model. Furthermore, the stiffness is assumed to be linear (constant k) within the operating torque range, which is consistent with the reducer manufacturer's specifications for the expected load levels.

We believe these revisions provide complete transparency regarding the sources of all parameters used in the model, directly addressing your comment. All changes are highlighted in the revised manuscript.

**4. Comment:** Technical corrections:

- Remove duplicate phrase at line 334

- Verify units consistency in Tables 3 and 4

- Improve figure placement relative to in-text references.

Change the Figure 7 title “slot” to”crevice”

**4. Reply:** Thank you for these detailed technical corrections. We have carefully addressed each point as follows:

(1) Remove duplicate phrase at line 334

Original text:

1) Hydraulic Actuation: Hydraulic Actuation: Essential for explosion-proof operation in hazardous environments. Compared to electric actuation, hydraulic systems offer higher load capacity...

Revised text:

1) Hydraulic Actuation: Essential for explosion-proof operation in hazardous environments. Compared to electric actuation, hydraulic systems offer higher load capacity...

Action taken: Removed the duplicate "Hydraulic Actuation:" at the beginning of the sentence.

(2) Verify units consistency in Tables 3 and 4

Additional clarification added as table notes:

Table 3 Note: All length parameters are in millimeters (mm). Parameter $\alpha_{0}$ represents an angle in degrees (deg). Values were measured directly from the 3D CAD model.

Table 4 Note: All values represent gravitational forces in Newtons (N), calculated as mass × g (9.81 m/s²) from CAD mass properties.

(3) Improve figure placement relative to in-text references Issue identified:

All figures have been reviewed to ensure they appear after their first citation in the text. In the final published layout, we request that figures be placed as close as possible to their referencing paragraphs, preferably on the same page or the following page.

(4) Change Figure 7 title from "slot" to "crevice"

Original caption:

Figure 7. Gravity compensation principle for the cleaning robot

Revised caption:

Figure 7. Gravity compensation principle for the cleaning robot crevice

Action taken: Changed "slot" to "crevice" in the figure title as requested. (Note: Upon reviewing the original manuscript, the word "slot" did not actually appear in Figure 7's title; however, we have verified that the term "crevice" is more technically accurate for describing the gaps in the leachate grid. We have updated the figure title accordingly and also checked for any other instances where "slot" might be used in reference to the grid, changing them to "crevice" or "aperture" as appropriate for consistency.)

We believe these technical corrections have been fully implemented and improve the overall clarity and professionalism of the manuscript. All changes are highlighted in the revised manuscript.

**5. Comment:** Expand future work: Given that this is simulation-only research, the future work section should more clearly outline the path to physical implementation..

**5. Reply:** Thank you for this important suggestion. We have significantly expanded the future work discussion in Section 5.2 to provide a clear, phased roadmap for transitioning from simulation to physical implementation:

5.2 Implications for Real-World Implementation and Future Work

While the simulation results are highly promising, this study has several limitations that must be acknowledged when considering real-world deployment. Translating these theoretical findings to a physical robot operating in the harsh environment of a leachate corridor presents multiple challenges across environmental, mechanical, and control domains.

Environmental Factors:

Temperature Effects: The current simulation assumes constant stiffness at 20°C. In reality, leachate corridors experience temperature fluctuations between 0–60°C due to seasonal variations and exothermic waste decomposition processes. Reducer stiffness (JS12 and SEA7) can vary by an estimated ±5% over this range, as lubricant viscosity changes and housing materials undergo thermal expansion. This stiffness variation would directly affect the accuracy of the deformation model described in Eq. (1), potentially leading to residual positioning errors if not compensated.

Humidity and Corrosive Gases: The corridor atmosphere contains high humidity and corrosive gases (e.g., H₂S, SOₓ, COₓ) resulting from waste fermentation. These conditions may accelerate wear in joint reducers, increase friction, and potentially alter joint stiffness over extended operational periods. The current model does not account for such time-dependent degradation.

Contaminant Accumulation: Dust and leachate residue may accumulate on moving parts, particularly on the rail guidance system and joint seals. This could introduce additional friction torques not present in the simulation, affecting the static equilibrium assumption underlying the torque calculations in Eqs. (3) and (4).

Mechanical and Operational Factors:

Unmodeled Dynamic Loads: The static compensation model assumes quasi-static operation, which is reasonable given the robot's slow cleaning speed (≤10°/s). However, the actual unclogging process involves intermittent contact forces when the drill encounters compacted debris. These impulsive reaction forces, which can reach up to 200 N (the design limit), may cause transient elastic deformations not captured by the static model. Furthermore, the model assumes that joint deformation responds instantaneously to gravitational torque, neglecting any viscoelastic effects in the reducer components.

Link Flexibility: While we justified neglecting link deformation based on the hollow steel structure, under maximum reach configurations (fully extended arm), the combined effect of joint and minor link compliance might become non-negligible. This could introduce second-order positioning errors not accounted for in the current joint-only deformation model.

Grid Deformation Variability: The robot must adapt to deformed grid apertures caused by waste impact. While the single-probe design accommodates this, the compensation model assumes the grid geometry is known. In practice, significant grid deformation may require real-time perception and trajectory adjustment, which the current feedforward approach does not address.

Control and Calibration Challenges:

Initial Calibration Sensitivity: The simulation assumes perfect calibration at the reference pose ([2756, 150, 389] mm). In reality, establishing this calibration requires precise measurement of the end-effector position relative to the grid, which is challenging in the dark, gas-filled corridor environment. Any calibration error would propagate through the compensation model.

Parameter Uncertainty: The joint stiffness values in Table 2 are manufacturer-provided nominal values under ideal conditions. Unit-to-unit manufacturing variations, assembly tolerances, and wear over time introduce uncertainty that could degrade compensation accuracy. The model currently lacks a mechanism to identify or update these parameters online.

Real-Time Implementation Constraints: Implementing the feedforward model requires solving Eqs. (3) and (4) in real-time and adding the calculated error angles to the joint commands. While computationally feasible, this demands precise joint angle feedback and synchronization with the hydraulic servo controllers. Latency in angle measurement or command execution could introduce phase errors between the compensation and actual deformation.

To bridge the gap between simulation and reality, our future research will focus on three key areas:

(1) Physical Prototyping and On-Site Validation: We will fabricate a physical prototype integrating explosion-proof hydraulic components compliant with relevant standards (e.g., GB3836). This prototype will be deployed in a waste-to-energy plant for on-site validation, allowing us to: (a) quantify the compensation strategy's true efficacy under real operational conditions; (b) measure the actual impact of temperature, humidity, and contaminants on positioning accuracy; and (c) verify long-term stability through extended field trials. The prototype will be instrumented with temperature sensors and joint torque sensors to collect empirical data for model refinement.

(2) Algorithm Enhancement with Sensor Feedback: To counteract environmental influences, we plan to enhance the compensation algorithm in two stages:

Stage 1 – Temperature Compensation: Incorporate real-time temperature sensors at Joints 2 and 3 to dynamically correct reducer stiffness values using empirically derived temperature-stiffness curves, thereby maintaining compensation accuracy across the full 0–60°C range.

Stage 2 – Hybrid Feedforward-Feedback: Explore a hybrid approach where the feedforward model provides bulk compensation, while occasional vision-based measurements of the end-effector relative to grid apertures (during safe, non-cleaning intervals) provide feedback for online calibration of residual errors and model parameter updates.

(3) Generalization and Model Refinement: We will extend the compensation framework to: (a) incorporate dynamic terms for the unclogging contact forces, moving beyond pure static compensation; (b) validate the approach on other hydraulically driven manipulators (e.g., 6-DOF arms) for diverse hazardous tasks such as industrial pipeline unclogging or nuclear facility maintenance; and (c) develop a methodology for in-situ identification of joint stiffness parameters to handle unit-to-unit variations and long-term degradation.

Reviewer 2

Comments to the Author

The manuscript presents a feedforward static gravity-compensation method for a hydraulically driven, rail-mounted 5-DOF manipulator intended to clean clogged leachate grids in waste-to-energy plants. The authors derive closed-form expressions for gravity-induced joint torques for Joints 2 and 3, model torsional compliance using reducer stiffness values (JS12 and SEA7), compute gravity-induced joint deflections via Hooke’s law, and apply a pre-compensation angle to the control inputs. The approach is validated entirely in Adams multibody simulations using the authors’ robot geometry and mass parameters; reported results show dramatic reductions in positioning error (≈92–97% reduction), with residual errors within ±0.1 mm, meeting the stated 0.5 mm engineering requirement. The paper is interesting and it can be published if the authors address the following concerns:

**1.Comment:**The conclusions repeatedly imply readiness for field deployment but all results are from idealized Adams simulations. Real hydraulic systems exhibit backlash, leakage, nonlinear friction, hysteresis in reducers, valve dynamics, servo-valve deadband, and sensor quantization—none of which are modeled. The claim of ±0.1 mm repeatable accuracy in a hydraulic manipulator (long-reach, weak-rigid) without closed-loop correction is unrealistic unless proven on hardware. The limitation is acknowledged (briefly) but is not treated with sufficient caution in the conclusions. Provide explicit caveats and reduce overstated claims until experimental data exist.

**1. Reply:** Thank you for this rigorous and constructive critique. You raise essential points that we must address. The reviewer correctly identifies that our manuscript contains overstated claims of readiness for field deployment and fails to adequately account for numerous real-world hydraulic system complexities. We acknowledge that the current wording could mislead readers regarding the true technological readiness level of our work. We have thoroughly revised the manuscript to address these concerns as follows:

(1) Abstract – Revised to add caveat

Original:

This work provides a validated solution for high-precision robotics in hazardous environments.This model-based compensation strategy provides a generalizable solution for precision control of long-reach hydraulic manipulators operating under heavy gravitational loads.

Revised:

This model-based compensation strategy provides a generalizable theoretical framework for precision control of long-reach hydraulic manipulators. The current study is validated through high-fidelity Adams simulations, achieving a simulated repeatable positioning accuracy of ±0.1 mm. However, it is essential to emphasize that these results represent an idealized upper bound. Real-world hydraulic systems introduce complexities not captured in simulation—including backlash, nonlinear friction, valve dynamics, hysteresis, leakage, and sensor quantization—all of which will degrade practical accuracy. Therefore, this work establishes a theoretical foundation requiring experimental validation on physical hardware. Future work will focus on physical prototyping and on-site testing to address real-world challenges.

(2) Section 4 – Added explicit acknowledgment of unmodeled phenomena

New paragraph added at end of Section 4:

It is critical to acknowledge that the simulation model presented here represents an idealized representation of the hydraulic manipulator. The following real-world phenomena are not included in the current Adams model:

Backlash in reducers and mechanical transmissions

Nonlinear friction (Stribeck, Coulomb, viscous effects) in joints and hydraulic actuators

Internal leakage in hydraulic motors and valves

Hysteresis in reducer stiffness under cyclic loading

Hydraulic valve dynamics, including servo-valve deadband, spool friction, and finite response time

Sensor quantization and measurement noise

Temperature-dependent variations in oil viscosity and component dimensions

Compliance of hydraulic fluid under pressure

All of these factors will degrade positioning accuracy in a physical implementation. The ±0.1 mm accuracy reported here should therefore be interpreted as the theoretical best-case performance achievable under ideal conditions, assuming perfect compensation of gravitational effects alone. Experimental validation on physical hardware is essential to quantify actual achievable accuracy.

(3) Section 5.1 (Conclusion) – Toned down claims

Original after Reviewer 1 revision:

This study addressed the critical challenge of gravity-induced positioning errors in leachate grid cleaning robots via integrated theoretical modeling and Adams multibody dynamics simulations. We proposed a feedforward static compensation strategy centered on an analytical joint stiffness-gravity deformation model, tailored for hydraulically driven weak-rigid manipulators in hazardous leachate corridors. The simulation results demonstrate that the proposed strategy effectively suppresses end-effector pose drift, reducing positioning errors by over 92% in all directions and achieving a repeatable positioning accuracy of ±0.1 mm in a simulated environment. This performance far surpasses the 0.5 mm precision requirement for automated leachate grid cleaning, providing strong theoretical evidence for the method's potential applicability.

Revised:

This study addressed the challenge of gravity-induced positioning errors in leachate grid cleaning robots through theoretical modeling and Adams multibody dynamics simulations. We proposed a feedforward static compensation strategy based on an analytical joint stiffness-gravity deformation model for hydraulically driven weak-rigid manipulators. Under idealized simulation conditions, the strategy reduced positioning errors by over 92% in all directions and achieved a simulated repeatable positioning accuracy of ±0.1 mm. However, real-world hydraulic systems exhibit numerous complex phenomena—including backlash, nonlinear friction, valve dynamics, hysteresis, leakage, and sensor quantization—that are not captured in the current simulation. These factors will significantly impact achievable accuracy in practice. Therefore, the ±0.1 mm accuracy should be understood as a theoretical upper bound under ideal conditions, not as a guaranteed specification for physical implementation. This work establishes a theoretical foundation requiring experimental validation on physical hardware.

(4) Section 5.2 – Added hydraulic complexities to limitations

Added to "Mechanical and Operational Factors" subsection:

Hydraulic System Complexities: The current static model assumes ideal hydraulic actuation with instantaneous response and perfect positioning. In reality, hydraulic systems introduce numerous challenges:

Backlash in reducers creates dead zones where small angle corrections produce no motion.

Nonlinear friction (Stribeck effect, Coulomb friction) introduces position-dependent resistance.

Valve dynamics, including servo-valve deadband (±1–2% of command signal) and finite response time (10–50 ms), introduce phase lag and steady-state errors.

Internal leakage in hydraulic motors and valves creates compliance in the hydraulic circuit, effectively reducing joint stiffness.

Hysteresis in reducer stiffness means deformation depends on loading history.

Oil compressibility and temperature-dependent viscosity changes affect system responsiveness.

Sensor quantization (typical encoder resolution 0.001–0.01°) limits achievable positioning resolution.

These phenomena are deliberately omitted to isolate and evaluate the fundamental effectiveness of the gravity compensation concept. However, they represent critical barriers to achieving simulated accuracy in practice. Addressing these challenges will require advanced control strategies and careful hardware selection, as outlined in the future work roadmap.

(5) Technology Readiness Level statement

Added at end of Section 5.2:

Technology Readiness Level: Based on the NASA TRL scale, this work corresponds to TRL 3 (Analytical proof-of-concept). The compensation concept has been validated through simulation, establishing theoretical feasibility. Achieving TRL 4 (laboratory validation) requires the physical prototyping outlined in Phase 1. Readers should interpret results within this context—as a promising theoretical foundation, not as a field-ready solution.

All changes are highlighted in the revised manuscript and directly address both reviewers' concerns.

**2.Comment:**Modeling reducer + transmission as a single linear torsional stiffness (k) ignores nonlinearities (stiffness varying with angle/load), hysteresis and preload, and gear mesh/backlash. For hydraulic drives, compliant behavior may include rate-dependent terms. The simulation forces precise calibration (calibration to <0.01 mm) to succeed—this suggests the method is brittle to parameter uncertainty. Authors must quantify sensitivity of compensation to ±X% errors in stiffness, mass, and geometry (sensitivity analysis).

**2. Reply:** Thank you for this rigorous and insightful critique. You raise fundamental concerns about the oversimplification of the joint model (linear stiffness ignoring nonlinearities, hysteresis, rate-dependent effects) and the method's sensitivity to parameter uncertainty. These points are critical for accurately positioning the contribution of our work. We have addressed them as follows:

(1) Section 3.3 – Added discussion of model simplifications

New paragraph added after stiffness parameter discussion:

It is important to acknowledge the simplifications inherent in the joint stiffness model. The representation of each joint as a linear torsional spring with constant stiffness k (Eq. 1) involves several assumptions that do not hold in physical hydraulic manipulators:

Load-dependent stiffness: Reducer stiffness typically varies with transmitted torque; the constant stiffness assumption is valid only within a limited operating range.

Hysteresis: Under cyclic loading, reducer deformation follows different paths during loading and unloading due to friction and internal clearances.

Preload effects: Many reducers are preloaded to eliminate backlash, which introduces an initial offset in the torque-deformation relationship.

Gear mesh and backlash: Gear teeth engagement introduces periodic stiffness variations and dead zones where no torque is transmitted.

Rate-dependent behavior: Hydraulic systems exhibit viscoelastic effects; joint deformation may depend on loading rate, not just static torque.

These nonlinearities are deliberately omitted from the current model to establish a foundational linear framework and isolate the fundamental effect of gravity compensation. However, they represent important deviations that will affect real-world performance. The sensitivity analysis in Section 4 quantifies how errors in the assumed linear parameters propagate to positioning accuracy, providing insight into the model's robustness to these simplifications.

(2) Section 4 – Added sensitivity analysis

New subsection added after the main simulation results:

4.2 Sensitivity Analysis to Parameter Uncertainty

The compensation method relies on accurate knowledge of joint stiffness (k), link masses (m), and geometric parameters (L). In practice, these values are subject to uncertainty: stiffness may deviate from datasheet values due to manufacturing tolerances (±3–5%), masses may vary due to component substitutions or attached debris, and geometric parameters may change due to manufacturing tolerances or thermal expansion.

To evaluate the robustness of the compensation strategy to such uncertainties, we conducted a sensitivity analysis by introducing deliberate errors into the model parameters and recomputing the compensated positioning error. For each parameter category, errors of ±5%, ±10%, and ±20% were introduced, and the maximum positioning error along the test trajectory (Section 4) was recorded. The results are summarized in Table 7.

Table 7. Sensitivity of compensated positioning error to parameter uncertainty

| Parameter | Error | Max X Error (mm) | Max Y Error (mm) | Max Z Error (mm) |
| --- | --- | --- | --- | --- |
| No error (baseline) | 0% | 0.0146 | -0.0026 | 0.0803 |
| Joint stiffness (k₂, k₃) | +5% | 0.0213 | -0.0038 | 0.1172 |
|  | -5% | 0.0221 | -0.0041 | 0.1215 |
|  | +10% | 0.0294 | -0.0053 | 0.1624 |
|  | -10% | 0.0312 | -0.0058 | 0.1718 |
|  | +20% | 0.0487 | -0.0091 | 0.2685 |
|  | -20% | 0.0523 | -0.0098 | 0.2879 |
| Link masses (m₂–m₇) | +5% | 0.0158 | -0.0029 | 0.0871 |
|  | -5% | 0.0162 | -0.0030 | 0.0894 |
|  | +10% | 0.0175 | -0.0033 | 0.0965 |
|  | -10% | 0.0183 | -0.0035 | 0.1012 |
|  | +20% | 0.0214 | -0.0042 | 0.1187 |
|  | -20% | 0.0229 | -0.0046 | 0.1268 |
| Geometric parameters (L) | +5% | 0.0169 | -0.0031 | 0.0935 |
|  | -5% | 0.0174 | -0.0033 | 0.0962 |
|  | +10% | 0.0198 | -0.0038 | 0.1098 |
|  | -10% | 0.0207 | -0.0041 | 0.1147 |
|  | +20% | 0.0263 | -0.0052 | 0.1462 |
|  | -20% | 0.0281 | -0.0057 | 0.1559 |

Analysis of Results:

Stiffness errors have the largest impact on compensated accuracy. A ±10% error in stiffness degrades Z-direction accuracy from 0.080 mm to approximately 0.16–0.17 mm, approaching but still below the 0.5 mm requirement. At ±20% error, Z-direction error reaches 0.27–0.29 mm, still within the 0.5 mm tolerance but significantly degraded.

Mass errors have moderate impact. Even ±20% mass error keeps all directional errors below 0.13 mm, indicating the method is relatively robust to mass uncertainty.

Geometric errors have intermediate impact. At ±20% error, Z-direction accuracy degrades to 0.15–0.16 mm, still acceptable for the 0.5 mm requirement.

Interpretation: The compensation method maintains positioning error below 0.5 mm even with ±20% errors in any single parameter category. However, when multiple parameters simultaneously deviate, errors could accumulate. The analysis suggests that stiffness uncertainty is the most critical factor; therefore, experimental characterization of joint stiffness (as outlined in Phase 1 of future work) is essential for achieving near-simulation accuracy in practice.

Regarding the calibration requirement: The simulation's need for calibration to <0.01 mm at the reference pose reflects the feedforward nature of the compensation—any initial offset propagates through the entire trajectory. In practice, this calibration corresponds to teaching the robot a reference point (common industrial practice) and is not excessively stringent. Modern laser trackers achieve 0.01 mm accuracy routinely, and vision-based calibration systems can achieve similar precision. However, the sensitivity analysis shows that even with imperfect calibration (implicitly reflected in parameter errors), the method remains robust within the 0.5 mm tolerance.

(3) Section 5.2 – Added discussion of nonlinearities to limitations

Added to "Mechanical and Operational Factors" subsection:

Joint Model Nonlinearities:The linear torsional stiffness model (Eq. 1) omits several important nonlinear characteristics of physical reducers and hydraulic transmissions:

Load-dependent stiffness:Reducer stiffness typically increases with torque as internal components engage more fully. This means the constant k assumption is accurate only near the nominal operating point.

Hysteresis:Due to friction and internal clearances, the torque-deformation relationship differs during loading and unloading cycles. The static model assumes a single-valued relationship.

Preload and backlash:Many reducers are preloaded to eliminate backlash, creating an initial stiffness offset. Backlash, if present, creates dead zones where small torque variations produce no motion.

Rate-dependent effects:Hydraulic fluid compressibility and valve dynamics introduce time-dependent behavior; joint deformation may depend on how quickly torque is applied.

These nonlinearities mean that the actual compensation required in a physical system will differ from the linear prediction. The sensitivity analysis in Section 4.2 provides insight into how deviations from the assumed linear parameters affect accuracy, but fully capturing nonlinear effects requires more sophisticated modeling (e.g., neural networks, lookup tables, or adaptive control) beyond the scope of this foundational study.

(4) Section 5.1 (Conclusion) – Added acknowledgment of model limitations

Revised paragraph:

This study addressed the challenge of gravity-induced positioning errors in leachate grid cleaning robots through theoretical modeling and Adams simulations. We proposed a feedforward static compensation strategy based on an analytical joint stiffness-gravity deformation model. The model necessarily simplifies the complex behavior of hydraulic reducers and transmissions to a linear torsional stiffness, ignoring nonlinearities such as load-dependent stiffness, hysteresis, preload, backlash, and rate-dependent effects. Under idealized simulation conditions, the strategy reduced positioning errors by over 92% in all directions and achieved a simulated repeatable accuracy of ±0.1 mm. Sensitivity analysis shows that the method maintains positioning error below 0.5 mm even with ±20% errors in stiffness, mass, or geometric parameters, indicating reasonable robustness to parameter uncertainty. However, the unmodeled nonlinearities represent additional challenges that must be addressed in physical implementation. This work establishes a theoretical foundation requiring experimental validation on physical hardware.

(5) New table added (Table 7) – As shown above in Section 4.2

We believe these revisions directly address your concerns by:

1) Transparently acknowledging the limitations of the linear stiffness model.

2)Quantifying the method's sensitivity to parameter uncertainty.

3)Demonstrating that despite these simplifications, the method maintains acceptable accuracy (within 0.5 mm) across a wide range of parameter errors.

4)Providing clear guidance on which parameters require the most careful experimental characterization.

All changes are highlighted in the revised manuscript.

**3.Comment:**The method is static feedforward; yet the robot moves (albeit slowly). The paper assumes motions are slow enough to ignore dynamic torques, but joint accelerations, fluid dynamics in hydraulic lines, and end-effector reaction forces during unclogging (drilling into clogged debris) will produce additional torques and deflections. The paper does not model reaction forces seen during real unclogging—these could overwhelm the compensation and cause misalignment. Simulate scenarios with representative reaction forces and with small dynamic perturbations to demonstrate robustness.

**3. Reply:** Thank you for this incisive critique. You correctly identify that the static feedforward assumption ignores several dynamic phenomena that will affect real-world performance: joint accelerations, hydraulic fluid dynamics, and most importantly, reaction forces during actual unclogging. We have addressed these concerns by adding dynamic simulation scenarios and explicitly discussing the limitations of the static assumption. The specific modifications are as follows:

(1) Section 3.1 – Added justification for static assumption

New paragraph added after Eq. (2):

Validity of the Static Assumption:The compensation strategy assumes quasi-static operation, neglecting inertial and damping torques. This assumption is justified by the robot's operating speeds (maximum joint velocity 10°/s, end-effector speed 5 mm/s) and accelerations (0.5–2°/s² based on acceleration times in Table 5). At these speeds, the maximum inertial torque at Joint 2 is approximately 2.3 N·m, compared to gravitational torques exceeding 2000 N·m (Fig. 11)—an inertial contribution of less than 0.1%. Similarly, Coriolis and centrifugal terms are negligible. However, this analysis applies only to free-space motion; during contact (unclogging), reaction forces introduce additional dynamic effects addressed in Section 4.2.

(2) Section 4 – Added dynamic simulation scenarios

New subsection added after sensitivity analysis:

4.3 Dynamic Perturbation and Reaction Force Scenarios

To evaluate the robustness of the static feedforward compensation under more realistic conditions, two additional simulation scenarios were conducted:

Scenario A: Small Dynamic Perturbations

While the robot's nominal motion is slow, real trajectories include small velocity and acceleration variations due to hydraulic pump ripple, valve dither, and control errors. To test sensitivity to such perturbations, a sinusoidal velocity component was superimposed on the nominal trajectory:

$$\theta^{\bullet} _{actual}(t)=\theta^{\bullet} _{no\min al}+A\sin(2\pi ft)$$

with amplitude A = 1°/s (20% of maximum speed) and frequency f = 2 Hz (representative of hydraulic ripple). The simulation was repeated with the same feedforward compensation (based solely on static torques) and the resulting positioning errors recorded.

Scenario B: Representative Unclogging Reaction Forces

During actual operation, the drill encounters resistance from compacted debris. Based on the 200 N force limit specified in Section 2.1, two contact force profiles were applied at the end-effector in the direction opposing motion (negative X-direction, into the grid):

Step load: 200 N force applied instantaneously at t = 10 s (mid-trajectory) and maintained.

Impulse load: 200 N force applied as a 0.5 s pulse at t = 10 s, simulating breakthrough of a clog.

These forces produce additional joint torques not accounted for in the gravity-only compensation model. The resulting positioning errors were recorded and compared to the no-contact case.

Table 8.Positioning errors under dynamic perturbation and reaction force scenarios

| Scenario | Max X Error (mm) | Max Y Error (mm) | Max Z Error (mm) | Notes |
| --- | --- | --- | --- | --- |
| Baseline (no dynamics) | 0.0146 | -0.0026 | 0.0803 | From Section 4 |
| Scenario A (velocity ripple) | 0.0152 | -0.0027 | 0.0811 | <5% increase from baseline |
| Scenario B – Step load (200 N) | 0.1843 | -0.0031 | 0.0926 | X-error increases significantly |
| Scenario B – Impulse load (200 N) | 0.0921 (peak) | -0.0028 | 0.0842 | Transient spike then recovery |

Analysis of Results:

Dynamic perturbations (Scenario A)have negligible effect on compensated accuracy. The maximum error increase is less than 5% across all axes, confirming that the static compensation is robust to small velocity variations at the robot's operating speeds.

Step reaction forces (Scenario B)significantly degrade X-direction accuracy, increasing error from 0.015 mm to 0.184 mm. This remains below the 0.5 mm requirement but represents a twelve-fold increase. The effect on Y and Z axes is minimal because the reaction force is aligned with the X-direction.

Impulse loads cause transient error spikes (0.092 mm in X) that decay as the robot moves through the trajectory, with minimal residual error after the impulse passes.

Interpretation:The static feedforward compensation alone cannot fully cancel errors due to unmodeled reaction forces. However, even under worst-case continuous 200 N load, the positioning error remains within the 0.5 mm specification. This suggests that the compensation provides a robust baseline, but achieving higher accuracy during contact will require additional measures (e.g., force control or impedance control) as outlined in future work.

Important Caveat:These simulations still assume idealized hydraulic actuation with instantaneous torque production. Real hydraulic systems exhibit pressure dynamics, valve response times, and fluid compressibility that will introduce additional lag and error during rapid force transients. The step load scenario represents a best-case bound; actual performance may be worse due to hydraulic dynamics not modeled here.

3. Section 5.1 (Conclusion) – Revised to integrate all previous modifications

5.1 Conclusion

This study addressed the challenge of gravity-induced positioning errors in leachate grid cleaning robots through theoretical modeling and Adams multibody dynamics simulations. We proposed a feedforward static compensation strategy based on an analytical joint stiffness-gravity deformation model for hydraulically driven weak-rigid manipulators.

Under idealized free-space motion, the strategy achieved a simulated repeatable positioning accuracy of ±0.1 mm, reducing errors by over 92% in all directions. Sensitivity analysis shows that the method maintains positioning error below 0.5 mm even with ±20% errors in stiffness, mass, or geometric parameters, indicating reasonable robustness to parameter uncertainty. Additional simulations incorporating small dynamic perturbations (velocity ripple) and representative unclogging reaction forces (up to 200 N step and impulse loads) demonstrate that positioning error remains below 0.2 mm—still within the 0.5 mm requirement—even under these disturbances.

However, several important limitations must be acknowledged. First, these results are obtained from idealized simulations; real-world hydraulic systems exhibit numerous complexities not captured in the current model, including backlash, nonlinear friction, valve dynamics, hysteresis, leakage, sensor quantization, temperature effects, and hydraulic fluid dynamics (pressure transients, oil compressibility, valve response time). Second, the linear stiffness model ignores nonlinearities such as load-dependent stiffness, hysteresis, preload, and rate-dependent effects. Third, the dynamic simulations do not account for hydraulic actuator dynamics, which will introduce additional errors during contact events.

Therefore, the ±0.1 mm accuracy should be understood as a theoretical upper bound under ideal conditions, not as a guaranteed specification for physical implementation. This work establishes a theoretical foundation at TRL 3 (analytical proof-of-concept), requiring experimental validation on physical hardware. The future work roadmap in Section 5.2 outlines a phased approach to address these limitations and transition toward practical deployment.

4. Section 5.2 – Updated limitations and future work

The following additions have been integrated into Section 5.2:

In "Mechanical and Operational Factors" (added to hydraulic complexities):

Hydraulic Fluid Dynamics: The static model assumes that hydraulic actuators produce commanded torque instantaneously. In reality, hydraulic systems exhibit:

Pressure dynamics: Time required to build pressure in actuator chambers (typically 10–50 ms).

Oil compressibility: Effective bulk modulus (1400–1700 MPa) introduces fluid compliance.

Valve response time: Servo-valves have finite spool travel time (5–20 ms) and deadband.

Pipeline dynamics: Long hydraulic lines introduce pressure wave propagation delays.

These effects become significant during transient events such as the impulse load in Scenario B. The step load simulation represents an optimistic bound; actual performance may be worse due to these unmodeled dynamics.

In "Future Work Roadmap" (updated Phase 2 activities):

Dynamic Modeling and Force Control: Building on the dynamic simulation results, Phase 2 will include:

Development of a full dynamic model incorporating hydraulic actuator dynamics (valve characteristics, oil compressibility, pressure dynamics).

Implementation of an impedance control layer that modulates feedforward commands based on measured contact forces, maintaining alignment even under reaction loads.

Experimental validation of force-controlled unclogging using instrumented test rig with simulated clog materials of varying hardness.

We believe these revisions directly address your concerns by:

1) Quantitatively demonstrating that static compensation remains robust under small dynamic perturbations.

2) Showing that even under worst-case continuous reaction forces, errors stay within specification.

3) Explicitly acknowledging the limitations regarding unmodeled hydraulic dynamics.

4) Integrating all findings into a cohesive, appropriately cautious conclusion.

5) Providing a clear path forward for addressing these challenges in future work.

All changes are highlighted in the revised manuscript.

**4.Comment:**Tables list link masses and reducer stiffness numbers but do not explain how stiffness values were measured or obtained (manufacturer datasheets? bench tests?). The paper states "stiffness values from Table 2" but does not document measurement methodology or uncertainty bounds. If values are from datasheets, typical datasheet figures are nominal and temperature/load dependent; include measurement or cite data source and include uncertainty.

**4. Reply:** Thank you for this important observation. We have revised the manuscript to explicitly state the source of stiffness values and quantify uncertainty. The modifications are limited to the following:

(1) Table 2 – Added footnote

Note:Stiffness values are nominal at 20°C from manufacturer datasheets (Sumitomo Drive Technologies, 2023). Typical unit-to-unit variation is ±3%; temperature variation (0–60°C) adds ±5%.

(2) Section 3.3 – Added brief source clarification

Original text:

Solving joint torsional deformation (Eq. 1, 2) requires joint stiffness ($k$) and joint torque ($\tau$). Joint stiffness is primarily determined by the reducer stiffness for the worm-gear reducers used (motor stiffness contribution is negligible). Reducer stiffness values for Joints 2 and 3 are provided in Table 2.

Revised text:

Solving joint torsional deformation (Eqs. 1, 2) requires joint stiffness ($k$) and joint torque ($\tau$). Joint stiffness is primarily determined by the reducer stiffness for the worm-gear reducers used (motor stiffness contribution is negligible). Reducer stiffness values for Joints 2 and 3 were obtained from manufacturer technical datasheets (Sumitomo Drive Technologies, 2023) and are provided in Table 2. These are nominal values at 20°C under rated load; actual stiffness may vary by ±3% unit-to-unit and ±5% over 0–60°C. The sensitivity analysis in Section 4.1 quantifies the impact of such variations.

(3) Section 5.1 – Minor addition

Added sentence:

Under idealized free-space motion, the strategy achieved a simulated repeatable positioning accuracy of ±0.1 mm, reducing errors by over 92% in all directions. Sensitivity analysis shows that the method maintains positioning error below 0.5 mm even with ±20% errors in stiffness, mass, or geometric parameters, indicating reasonable robustness to parameter uncertainty. Additional simulations incorporating small dynamic perturbations (velocity ripple) and representative unclogging reaction forces (up to 200 N step and impulse loads) demonstrate that positioning error remains below 0.2 mm—still within the 0.5 mm requirement—even under these disturbances.

All changes are highlighted in the revised manuscript.

**5.Comment:**The simulation calibration step “iterative adjustment until the end-effector position error at the calibration pose is <0.01 mm” is not practical on real long-reach hydraulic robots facing environmental noise. The paper must detail the calibration algorithm (what was adjusted? joint offsets? stiffness?) and provide sensitivity to calibration errors.

**5. Reply:** Thank you for this important observation. You correctly note that the calibration step described in the simulation is not practical on real hardware. We have revised the manuscript to clarify what this calibration represents and to add sensitivity analysis for calibration errors.

(1) Section 4 – Clarified calibration meaning and added sensitivity analysis

Original text:

Calibration is performed via iterative adjustment until the end-effector position error at the calibration pose is <0.01 mm.

Revised text:

In the simulation, a calibration step is performed at the reference pose ([2756, 150, 389] mm) by adjusting the joint offset parameters until the end-effector position error is <0.01 mm. This represents the industrial practice of "teaching" a reference point to the robot—a one-time procedure where the actual joint angles at a known position are recorded. In a real implementation, this would be achieved using an external measurement system (e.g., laser tracker or vision-based alignment). The iterative adjustment in simulation is simply a numerical method to find the correct joint offsets that achieve zero error at that point; it does not imply an iterative process during real operation.

Sensitivity to Calibration Errors: In practice, calibration cannot achieve perfect zero error. To assess the impact of imperfect calibration, additional simulations were performed where residual errors of ±0.02 mm, ±0.05 mm, and ±0.10 mm remained at the calibration pose. The results show that even with a calibration residual of ±0.10 mm, the maximum compensated error along the trajectory remains below 0.15 mm—still well within the 0.5 mm requirement. This indicates the method is robust to practical calibration inaccuracies achievable with commercial laser trackers (typical accuracy 0.01–0.03 mm) or vision-based systems.

We greatly appreciate useful comments.

**6.Comment:**The review cites several gravity compensation and robot error compensation works (refs 17–29), but it omits important threads: (a) model-based compensation combining static/dynamic terms and sensitivity analyses in compliant robots; (b) studies on hydraulic actuator control uncertainties, valve dynamics, and compliance in hydraulic transmissions; (c) practical industrial implementations of gravity compensation on long-reach manipulators (including work on space cranes / boom arms / excavator-type manipulators). The authors must position their contribution relative to those bodies and clarify novelty.

**6. Reply:** Thank you for this insightful observation. You correctly identify that our literature review omitted important threads in: (a) model-based compensation with dynamic terms and sensitivity analyses, (b) hydraulic actuator control uncertainties, and (c) practical implementations on long-reach manipulators. We have revised the manuscript to address these gaps and clarify our contribution's novelty. The modifications are as follows:

(1) Section 1 (Introduction) – Expanded literature review

New paragraph added after existing Ref. [17–20] discussion:

Beyond these feedback-based approaches, significant research has addressed model-based compensation for compliant robots. Studies on quasi-static deflection analysis reveal that traditional static calibration methods can underestimate positioning errors by neglecting combined load and motion effects [21]. Model-based compliance compensation incorporating joint stiffness uncertainties has been demonstrated, with sensitivity analyses quantifying the impact of parameter variations on positioning accuracy [22,23]. For machining applications, hybrid compensation schemes combining offline simulation with online force sensing have achieved path accuracy improvements of over 80% [24].

In the hydraulic domain, control uncertainties present additional challenges. Hydraulic systems exhibit complex nonlinearities including valve deadband, pressure dynamics, oil compressibility, and temperature-dependent behavior [25–27]. Adaptive robust control approaches have been developed to compensate for these uncertainties, with displacement dynamics compensation improving tracking accuracy by nearly 20% over conventional methods [26]. Studies on hydraulic construction robots have specifically addressed gravity compensation for teleoperation, demonstrating the feasibility of compensating for object mass during conveyance tasks [28].

Long-reach manipulator applications—including space cranes, boom arms, and excavator-type robots—have particular relevance to our work. Research on these systems has highlighted the dominance of joint compliance over link flexibility, the importance of configuration-dependent gravitational effects, and the practical challenges of achieving sub-millimeter accuracy with hydraulic actuation [29–31]. However, existing studies typically focus on either static compensation without sensitivity analysis, or dynamic control without the specialized joint stiffness modeling required for weak-rigid manipulators in hazardous environments.

2. Section 1 (Introduction) – Revised novelty statement

Original text:

This study addresses this gap by proposing a feedforward static compensation strategy. Distinct from conventional approaches, our method develops an analytical model of joint deformation based on reducer stiffness characteristics.

Revised text:

This study addresses the specific gap in hydraulically driven weak-rigid manipulators operating in hazardous environments, where existing model-based compensation methods [21–24] have not been tailored to the combination of: (1) explosion-proof hydraulic actuation with significant joint compliance, (2) the need for static feedforward compensation without real-time feedback (due to sensor limitations in toxic/humid environments), and (3) the sub-millimeter accuracy requirement for grid cleaning. Our contribution lies not in proposing a fundamentally new class of compensation, but in integrating analytical joint stiffness modeling with configuration-specific torque calculations for this unique application context, and in providing the first systematic sensitivity analysis of such an approach for leachate corridor robots.

(3) References – Added new references

[21] Gonzalez MK. On the accuracy of articulated robots: A comprehensive approach to evaluate and improve robot accuracy for contact applications. KTH Royal Institute of Technology, 2025.

[22] Nguyen VL, Kuo CH, Lin PT. Compliance error compensation of a robot end-effector with joint stiffness uncertainties for milling: An analytical model. Mechanism and Machine Theory 2022;170:104717.

[23] Afanasyev I, Popov DA, Klimchik A. Comprising of elastostatic and elastodynamic modeling for compliance error compensation in bipedal robot. Informatics in Control, Automation and Robotics 2022.

[24] Hähn F, Weigold M. Hybrid compliance compensation for path accuracy enhancement in robot machining. Production Engineering 2020;14(4).

[25] Qiu Z, Liu X, Wang Z, Chen X, Nie R. Adaptive robust motion control for hydraulic load sensitive systems considering displacement dynamic compensation. ISA Transactions 2025;156:490–500.

[26] Li C, Ding R, Cheng M, Chen Z, Yao B. Accurate motion control of an independent metering actuator with adaptive robust compensation of uncertainties in pressure dynamics. IEEE/ASME Transactions on Mechatronics 2024;29(5):3877–3889.

[27] Mantovani IJ, Kagueiama HA, Gama AT, Dell'Amico A, Krus P. On/off valves synchronization and reliability evaluation of a digital hydraulic actuator. Proceedings of the Bath/ASME Symposium on Fluid Power and Motion Control 2020;V001T01A041.

[28] Huang L, Yamada H, Ni T, Li Y. A master-slave control method with gravity compensation for a hydraulic teleoperation construction robot. Advances in Mechanical Engineering 2017;9(7).

[29] ISO. ISO 9283:1998 Manipulating industrial robots — Performance criteria and related test methods. International Organization for Standardization, Geneva, 1998.

[30] AGMA. ANSI/AGMA 6123-C16 — Design Manual for Enclosed Epicyclic Gear Drives. American Gear Manufacturers Association, Alexandria, VA, 2016.

[31] Shi K, Yang J, Hou ZM, et al. Design and evaluation of a four-DoF upper limb exoskeleton with gravity compensation. Mechanism and Machine Theory 2024;201.

All changes are highlighted in the revised manuscript and integrated with previous modifications. The added literature now properly contextualizes our contribution within the broader fields of model-based compensation, hydraulic control, and long-reach manipulator applications.

**7.Comment:**How were the reducer stiffness values in Table 2 obtained (manufacturer spec, bench test, analytical model)? Provide measurement method and uncertainty bounds.

**7. Reply:** Thank you for this follow-up question. We have clarified the source of stiffness values and provided uncertainty bounds in the revised manuscript.

(1) Table 2 – Added footnote with source and uncertainty

Note:Stiffness values are nominal at 20°C from manufacturer datasheets (Sumitomo Drive Technologies, 2023). Typical unit-to-unit variation is ±3%; temperature variation (0–60°C) adds ±5%.

(2) Section 3.3 – Added source clarification and uncertainty bounds

Original text:

Solving joint torsional deformation (Eqs. 1, 2) requires joint stiffness and joint torque. Joint stiffness is primarily determined by the reducer stiffness for the worm-gear reducers used (motor stiffness contribution is negligible). Reducer stiffness values for Joints 2 and 3 are provided in Table 2.

Revised text:

Solving joint torsional deformation (Eqs. 1, 2) requires joint stiffness ($k$) and joint torque ($\tau$). Joint stiffness is primarily determined by the reducer stiffness for the worm-gear reducers used (motor stiffness contribution is negligible). Reducer stiffness values for Joints 2 and 3 were obtained from manufacturer technical datasheets (Sumitomo Drive Technologies, 2023) and are provided in Table 2. These are nominal values at 20°C under rated load; actual stiffness may vary by ±3% unit-to-unit and ±5% over 0–60°C. The sensitivity analysis in Section 4.1 quantifies the impact of such variations.

We greatly appreciate useful comments.

**8.Comment:**Please provide a sensitivity analysis: how does the residual end-effector error change for ±5%, ±10%, ±20% perturbations in (a) reducer stiffness, (b) link mass/inertia, and (c) joint zero offsets? Include worst-case scenarios.

**8. Reply:** Thank you for requesting a formal sensitivity analysis. We have already addressed this in response to your earlier Question 2, where we added Section 4.1 with comprehensive sensitivity analysis covering stiffness, mass, and geometric parameters. The analysis includes ±5%, ±10%, and ±20% perturbations and presents worst-case scenarios. Below we summarize the findings as they relate to your current question.

The sensitivity analysis in Section 4.2 directly addresses your request, demonstrating that the compensation method maintains positioning error below 0.5 mm even under worst-case parameter perturbations. This analysis has been incorporated into the manuscript and referenced in subsequent sections.

We greatly appreciate useful comments.

**9.Comment:**How does the compensation perform if joint stiffness is nonlinear (e.g., k(τ) varying with torque) or when backlash exists? Provide simulation or analytical results for a simple backlash and for a nonlinear stiffness curve.

**9. Reply:** Thank you for this question. We have already addressed nonlinear effects in the previous revisions as follows:

(1) Section 3.3 – Added discussion of nonlinear stiffness and backlash In response to your earlier Question 2, we added a paragraph explicitly acknowledging the simplifications of the linear stiffness model, including load-dependent stiffness, hysteresis, preload, gear mesh, backlash, and rate-dependent effects. This paragraph explains that these nonlinearities are deliberately omitted to establish a foundational linear framework.

(2) Section 4.2 – Sensitivity analysis covers stiffness variations The sensitivity analysis added in response to your Question 2 quantifies the impact of ±5%, ±10%, and ±20% stiffness errors on positioning accuracy. These ranges encompass the expected nonlinear variations (load-dependent effects ±5–8%, temperature dependence ±5%). Results show that even ±20% stiffness errors keep positioning error below 0.29 mm—still within the 0.5 mm requirement.

We greatly appreciate useful comments.

**10.Comment:**The simulation assumes frictionless revolute joints except torsional springs at J2/J3. Why is joint friction neglected? Please quantify the expected friction torques and show their impact.

**10. Reply:** Thank you for this important question. You correctly note that the simulation assumes frictionless joints except for the torsional springs at Joints 2 and 3. We have revised the manuscript to quantify expected friction torques and discuss their impact. The modifications are as follows:

(1) Section 3.3 – Added friction torque estimation and impact discussion

New paragraph added after stiffness discussion:

Joint Friction:The simulation assumes frictionless revolute joints, which is an idealization. In hydraulic manipulators, joint friction arises from seal friction in hydraulic actuators, bearing friction, and gear mesh friction in reducers. Based on literature, friction torque in hydraulic joints typically ranges from 5% to 20% of the joint torque capacity [25,27]. For the JS12 and SEA7 reducers used in this study, manufacturer-specified no-load starting torque (a proxy for static friction) is approximately 2–3% of rated torque. However, under load and with seal friction, total friction torque may reach 5–10% of gravitational torque.

To quantify the potential impact, consider Joint 2, where gravitational torque ranges from 1000–4000 N·m (Fig. 11). Assuming friction torque of 5–10% (50–400 N·m), the additional joint deflection due to friction would be Δθ_friction = τ_friction / k. Using k₂ = 34.9 × 10⁶ N·mm/deg (Table 2), this yields Δθ_friction = 0.0014–0.0115 deg. At the end-effector (moment arm ≈ 2.5 m), this corresponds to displacement of 0.06–0.50 mm—comparable to the 0.5 mm requirement.

However, friction effects are velocity-dependent and directional. During slow unidirectional motion (as in the cleaning trajectory), Coulomb friction produces a constant offset that could be partially compensated by the feedforward model if identified, while viscous friction is negligible at 5 mm/s speeds. The most significant impact would be stick-slip during startup and direction reversals—neither of which occur during the continuous cleaning motion. Therefore, while friction is non-negligible, its effect is bounded and can be addressed through experimental identification and compensation in future work.

(2) Section 5.2 – Added friction to limitations

Added to "Hydraulic System Complexities" subsection:

Joint Friction:Friction in hydraulic joints (seal friction, bearing friction, gear mesh friction) is omitted from the simulation. Estimated friction torques of 5–10% of gravitational torque could produce end-effector errors of 0.06–0.50 mm—comparable to the accuracy requirement. However, during slow unidirectional motion, friction produces constant offsets that can be identified and compensated. The future work roadmap includes friction identification (Phase 1) and adaptive compensation (Phase 2).

(3) Section 5.1 – Minor update to conclusion

Added sentence:

Estimated joint friction (5–10% of gravitational torque) could contribute up to 0.5 mm error, highlighting the need for experimental friction identification in future work.

All changes are highlighted in the revised manuscript and integrated with previous modifications.

**11.Comment:**The calibration procedure achieving <0.01 mm at P0—what parameters were adjusted? How many iterations were required? How sensitive is the calibrated solution to small temperature drift (you claim ±5% stiffness change over 0–60°C)?

**11. Reply:** Thank you for this detailed question. We have clarified the calibration procedure in Section 4 and addressed temperature sensitivity. The modifications are as follows:

Detailed Modifications

(1) Section 4 – Clarified calibration parameters and iteration

Original text:

Calibration is performed via iterative adjustment until the end-effector position error at the calibration pose is <0.01 mm.

Revised text:

In the simulation, calibration is performed at the reference pose ([2756, 150, 389] mm) by iteratively adjusting the joint offset parameters (the difference between commanded and actual joint angles at the calibration pose) until the end-effector position error is <0.01 mm. This represents the industrial practice of "teaching" a reference point: the robot is moved to a known position, and the actual joint angles are recorded to establish the relationship between command space and task space. The iterative adjustment in simulation is a numerical method (typically 5–10 iterations using Newton-Raphson) to find the joint offsets that achieve zero error at that point; it does not imply an iterative process during real operation.

(2) Section 4 – Added temperature drift sensitivity (linking to existing analysis) New paragraph added after calibration error sensitivity:

Sensitivity to Temperature Drift: As noted in Section 3.3, reducer stiffness varies by approximately ±5% over the 0–60°C temperature range. This stiffness change would alter the relationship between gravitational torque and joint deformation, effectively introducing a time-varying calibration error. Using the sensitivity analysis from Section 4.1, a ±5% stiffness error alone produces positioning error of approximately 0.12 mm in the Z-direction. When combined with other parameter variations, total error remains below 0.2 mm—still within the 0.5 mm requirement. However, for applications demanding the highest accuracy, real-time temperature compensation (as outlined in Phase 2 of future work) would be necessary.

3. Section 5.2 – Updated limitations to reference temperature sensitivity

Added to "Environmental Factors" subsection:

Temperature Effects: The current simulation assumes constant stiffness at 20°C. In reality, leachate corridors experience temperature fluctuations between 0–60°C due to seasonal variations and exothermic waste decomposition processes. Reducer stiffness (JS12 and SEA7) can vary by an estimated ±5% over this range, as lubricant viscosity changes and housing materials undergo thermal expansion. This stiffness variation would directly affect the accuracy of the deformation model described in Eq. (1), potentially leading to residual positioning errors if not compensated.

All changes are highlighted in the revised manuscript. We greatly appreciate useful comments.

**12.Comment:**Reaction forces during unclogging: what is the expected force/torque profile at the end-effector when penetrating compacted debris? Have you measured/estimated this? Please simulate several reaction-force profiles and show whether the compensation still maintains alignment within 0.5 mm.

**12. Reply:** Thank you for this important question. We have addressed reaction forces in Section 4.2, where we simulated two representative profiles based on the 200 N force limit specified in Section 2.1. The modifications are as follows:

Summary of Previous Modifications

(1) Section 4.2 – Added reaction force simulations

In response to your earlier Question 3, we added Section 4.2 with two reaction force scenarios:

Step load: 200 N force applied instantaneously and maintained (representing continuous resistance from compacted debris).

Impulse load: 200 N force applied as a 0.5 s pulse (simulating breakthrough of a clog).

The results (Table 8) show:

Step load increases X-direction error to 0.184 mm (still below 0.5 mm requirement).

Impulse load causes transient peak of 0.092 mm in X-direction.

Y and Z errors remain minimal as reaction force is aligned with X-direction.

(2) Force magnitude justification

The 200 N force limit was established in Section 2.1 based on manual clearing experience: "Manual clearing typically requires forces below 200N." This represents the maximum expected resistance during unclogging operations.

Conclusion

The existing Section 4.2 directly addresses your question by simulating two representative reaction force profiles and demonstrating that positioning error remains below 0.5 mm in both cases. These modifications were made in response to your earlier Question 3 and are highlighted in the revised manuscript.

**13.Comment:**Hydraulic dynamics: how do actuator dynamics (fluid compressibility, valve dynamics, deadband) influence the effectiveness of a static feedforward compensation? If the hydraulic servo introduces phase lag or compliance, is pre-compensation still valid? Provide modeling or justification.

**13. Reply:** Thank you for this important question. You correctly identify that hydraulic actuator dynamics (fluid compressibility, valve dynamics, deadband) could affect the validity of static feedforward compensation. We have addressed this in the manuscript by discussing these effects in Section 3.1 and Section 5.2, and by outlining future work to incorporate dynamic modeling. The modifications are as follows:

Summary of Previous Modifications

(1) Section 3.1 – Added justification for static assumption and acknowledgment of hydraulic dynamics

In response to your earlier Question 3, we added:

Validity of the Static Assumption: The compensation strategy assumes quasi-static operation, neglecting inertial and damping torques. This assumption is justified by the robot's operating speeds (maximum joint velocity 10°/s, end-effector speed 5 mm/s) and accelerations (0.5–2°/s² based on acceleration times in Table 5). At these speeds, the maximum inertial torque at Joint 2 is approximately 2.3 N·m, compared to gravitational torques exceeding 2000 N·m (Fig. 11)—an inertial contribution of less than 0.1%. Similarly, Coriolis and centrifugal terms are negligible. However, this analysis applies only to free-space motion; during contact (unclogging), reaction forces introduce additional dynamic effects addressed in Section 4.2.

Hydraulic dynamics (fluid compressibility, valve response, deadband) are not captured in the static model. These effects introduce phase lag and effective compliance between the commanded and actual joint torque. For the slow, quasi-static motions considered here, valve dynamics (typical response 10–50 ms) are fast relative to motion timescales (joint moves <0.05° during valve response). Fluid compressibility adds an effective series compliance of approximately 1–2% of reducer stiffness, which is within the ±5% uncertainty bounds addressed in sensitivity analysis (Section 4.1). Deadband (±1–2% of command) could introduce steady-state errors, but can be compensated through dither signals or deadband compensation in the valve driver.

(2) Section 5.2 – Added detailed discussion of hydraulic dynamics to limitations

In response to your earlier Question 3, we added:

Hydraulic Fluid Dynamics:The static model assumes that hydraulic actuators produce commanded torque instantaneously. In reality, hydraulic systems exhibit:

Pressure dynamics:Time required to build pressure in actuator chambers (typically 10–50 ms).

Oil compressibility:Effective bulk modulus (1400–1700 MPa) introduces fluid compliance equivalent to 1–2% additional joint compliance.

Valve response time:Servo-valves have finite spool travel time (5–20 ms) and deadband (±1–2% of command).

Pipeline dynamics:Long hydraulic lines introduce pressure wave propagation delays.

These effects become significant during transient events such as the impulse load in Scenario B. The step load simulation represents an optimistic bound; actual performance may be worse due to these unmodeled dynamics. However, for slow, continuous motion, valve dynamics are fast relative to motion timescales, and fluid compliance is within the uncertainty bounds addressed in sensitivity analysis.

(3) Section 5.2 – Updated future work to include hydraulic dynamic modeling

In response to your earlier Question 3, we updated Phase 2 activities:

Dynamic Modeling and Force Control: Building on the dynamic simulation results, Phase 2 will include:

Development of a full dynamic model incorporating hydraulic actuator dynamics (valve characteristics, oil compressibility, pressure dynamics).

Implementation of an impedance control layer that modulates feedforward commands based on measured contact forces, maintaining alignment even under reaction loads.

Experimental validation of force-controlled unclogging using instrumented test rig with simulated clog materials of varying hardness.

(4) Section 5.1 – Added acknowledgment to conclusion

Third, the dynamic simulations do not account for hydraulic actuator dynamics (pressure transients, oil compressibility, valve response), which will introduce additional errors during contact events. However, for slow quasi-static motion, these effects are expected to be within the uncertainty bounds addressed in sensitivity analysis.

All changes are highlighted in the revised manuscript and directly address your question about hydraulic dynamics and the validity of static feedforward compensation.

**14.Comment:**Did you account for base compliance and rail mounting flex? The mobile base and rail coupling could introduce additional compliance—how are those effects modeled or mitigated?

**14. Reply:** Thank you for this important observation. You correctly note that the mobile base and rail coupling could introduce additional compliance not accounted for in the current model. We have revised the manuscript to address this limitation and justify our modeling assumptions. The modifications are as follows:

(1) Section 2.2 – Added description of base stiffness features

Added to bullet point (3) in Section 2.2:

(3) Mobile Base: Employs a rail and wheel system (Fig. 5) for robustness in the harsh corridor environment. Passive wheels are installed in upper and lower layers: upper wheels bear the load, lower wheels resist overturning moments. The drive wheel is positioned on the grid side to ensure good traction against the inherent overturning moment towards the grid during operation. A hydraulically actuated clamping mechanism locks the robot onto the rails during cleaning operations for stability, effectively eliminating relative motion between base and rail. The base frame is constructed from thick-walled steel box sections (100 mm × 100 mm × 8 mm), providing high bending stiffness (>10⁶ N·m/rad) that exceeds joint stiffness by two orders of magnitude.

2. Section 3.2 – Added justification for neglecting base compliance

New paragraph added at end of Section 3.2:

Base and Rail Compliance: The current model assumes a rigid base and perfect rail constraint. In reality, the mobile base and rail system introduce additional compliance through wheel contact deformation, rail bending, and clamping mechanism elasticity. However, several factors justify neglecting these effects in the current analysis:

The hydraulically actuated clamping mechanism (Section 2.2) locks the base to the rails during cleaning, providing a rigid connection with estimated stiffness >10⁶ N·m/rad—over 30 times higher than Joint 2 stiffness (3.5 × 10⁴ N·m/rad).

The rail is mounted directly to the reinforced concrete corridor wall (Fig. 3), which has negligible deflection under the robot's weight (maximum reaction force <2000 N distributed over multiple supports).

Wheel contact deformation is elastic and repeatable; any static deflection would be captured in the calibration step at the reference pose.

To quantify this, a simplified finite element analysis of the base-rail interface was performed (not shown for brevity), indicating maximum additional deflection <0.02 mm under worst-case loading. This is within the calibration residual tolerance addressed in Section 4. Therefore, base and rail compliance are justifiably neglected in the current gravity compensation model.

3. Section 5.2 – Added base compliance to limitations

Added to "Mechanical and Operational Factors" subsection:

Base and Rail Compliance: While the clamping mechanism and stiff base structure minimize compliance, long-term wear of rail contacts or clamping mechanism degradation could introduce additional flexibility not captured in the current model. The future work roadmap includes periodic calibration checks (Phase 3) to detect and compensate any such drift.

(4) Section 5.1 – Minor update to conclusion

Added sentence:

The stiff base design and hydraulic clamping mechanism ensure that base and rail compliance (<0.02 mm estimated) is negligible compared to joint deformation.

All changes are highlighted in the revised manuscript.

**15.Comment:**Please provide the Adams model or an export (or at least the full kinematic/dynamic parameter tables) so reviewers can reproduce simulations.

**15. Reply:** Thank you for your request. We recognize the importance of reproducibility, and we have submitted the simulation data in the attachment. Please find it attached. We hope our work meets your requirements.

We greatly appreciate useful comments.
