# Supplementary material for: Gravity compensation for leachate grid cleaning robots in waste-to-energy plants: A modeling and simulation study
Source: PLoS One. 2026 May 19;21(5):e0349592. doi: 10.1371/journal.pone.0349592 (PMC13186384; doi:10.1371/journal.pone.0349592)
Supplement: Attachment — Submitted filename: Response_to_Reviewers_auresp_2.docx [file pone.0349592.s003.docx]

Response to reviewers

Dear Academic Editor: Shamshad Alam, PhD, Dear reviewers

We gratefully thank the Editor and all Reviewers for their time spent making constructive remarks and useful suggestions, which have significantly improved the quality of the manuscript. Each suggested revision and comment brought forward by the reviewers was accurately incorporated and considered. The Reviewers' comments are addressed below point by point with corresponding revisions.

Reviewer 1

Comments to the Author

The authors provided a comprehensive and detailed response to all review comments, significantly improving the scientific rigor and transparency of the manuscript.

Minor Issues Remaining:

**1.Comment:**Data Availability Statement: It is recommended to explicitly state the method of data access (e.g., providing Adams model files as supplementary material).

**1. Reply:** Thank you for your suggestion. We have revised the Data Availability Statement to explicitly state the method of data access. The Adams model files are now provided as supplementary material. The modification is as follows:

**Original:**

All data underlying the findings described in this manuscript are fully available within the paper. No external data repositories or supplementary files are required.

**Revised:**

All data underlying the findings described in this manuscript are fully available within the paper and in the supplementary material. The Adams simulation model files (including geometry, joint parameters, and simulation setup) are provided as S1 File accompanying this submission.

**2. Comment:** Completeness of Sensitivity Analysis: It is necessary to confirm that the results of Section 4.2 are complete.

**2. Reply:** Thank you for your request to confirm the completeness of the sensitivity analysis in Section 4.2. We have verified that Section 4.2 presents a comprehensive sensitivity analysis covering all relevant parameters. The details are as follows:

**Section 4.2 (Sensitivity Analysis to Parameter Uncertainty) includes:**

(1)Three parameter categories analyzed:

①Joint stiffness (k₂, k₃) – with perturbations of ±5%, ±10%, ±20%

②Link masses (m₂–m₇) – with perturbations of ±5%, ±10%, ±20%

③Geometric parameters (L) – with perturbations of ±5%, ±10%, ±20%

(2)Complete results table (Table 7) showing:

①Maximum X, Y, and Z positioning errors for each perturbation level

②Baseline (0% error) for comparison

③Both positive and negative perturbations for all categories

(3)Detailed analysis of results:

①Identification that stiffness errors have the largest impact

②Quantification that ±20% stiffness errors keep Z-error below 0.29 mm (within 0.5 mm requirement)

③Assessment that mass errors have moderate impact

④Evaluation that geometric errors have intermediate impact

(4)Interpretation of findings:

①Method maintains positioning error below 0.5 mm even with ±20% errors in any single parameter

②Stiffness uncertainty identified as most critical factor

③Recommendation for experimental characterization of joint stiffness

④Discussion of calibration requirements and robustness

**Conclusion**

The sensitivity analysis presented in Section 4.2 is complete and comprehensive, covering all relevant parameters with appropriate perturbation ranges and providing thorough analysis of results. No additional modifications are required.

We appreciate your careful review of our manuscript.

**3. Comment:** Figure 7 Caption: The phrasing is slightly awkward and needs adjustment.

**3. Reply:** Thank you for your observation regarding the phrasing of Figure 7 caption. We agree that the wording can be improved for clarity and natural flow. We have revised the caption as follows:

**Original:**

Figure 7. Gravity compensation principle for the cleaning robot crevice

**Revised:**

Figure 7. Gravity compensation principle for the cleaning robot operating near a grid crevice

This revision makes the caption more natural and clearly describes what the figure illustrates. The change is highlighted in the revised manuscript.
